# Comprehensive three-dimensional free-breathing magnetic resonance imaging for simultaneous myocardial viability and coronary artery visualization at 1.5T and 3T

**DOI:** 10.1016/j.jocmr.2025.102672

**Published:** 2025-12-12

**Authors:** Dongyue Si, Simon J. Littlewood, Michael G. Crabb, Karl P. Kunze, Claudia Prieto, René M. Botnar

**Affiliations:** a School of Biomedical Engineering and Imaging Sciences, King’s College London, London, UK; b MR Research Collaborations, Siemens Healthcare Limited, Camberley, UK; c School of Engineering, Pontificia Universidad Católica de Chile, Santiago, Chile; d Millennium Institute for Intelligent Healthcare Engineering, Santiago, Chile; e Institute for Biological and Medical Engineering, Pontificia Universidad Católica de Chile, Santiago, Chile; f Institute for Advanced Study, Technical University of Munich, Garching, Germany

**Keywords:** Late gadolinium enhancement, Coronary magnetic resonance angiography, 3D whole-heart, Coronary artery disease, Multi-contrast

## Abstract

**Background:**

Cardiovascular magnetic resonance is promising for non-invasive assessment of various cardiac diseases with the ability to provide multi-contrast images, including late gadolinium enhancement (LGE) for myocardial tissue characterization and coronary magnetic resonance angiography (CMRA) for anatomical imaging. However, LGE and CMRA are usually acquired separately in clinical routine with unmatched spatial resolution and slice positions. In this proof of concept study, we aim to achieve a one-stop imaging of 3D gray-blood phase-sensitive inversion recovery (PSIR) LGE and 3D CMRA by proposing a free-breathing simultaneous Gray-Blood and Bright-blOOd phase SensiTive inversion recovery (GB-BOOST) sequence.

**Methods:**

The proposed research sequence acquires two interleaved 3D volumes with inversion recovery and T2 preparation pulses to obtain gray-blood PSIR and CMRA, respectively. Two-dimensional image navigator (iNAV) is performed before the acquisition of each volume to detect respiratory motion, enabling free-breathing acquisition with 100% respiratory scan efficiency. The GB-BOOST framework is compatible with both Dixon gradient echo (GRE) and balanced steady-state free precession (bSSFP) sequences for the application at 3T and 1.5T. In-vivo validation experiments included in total 23 patients for GB-BOOST, which were performed on either a 3T or a 1.5T clinical scanner. The performance of the proposed sequence was compared with clinical 2D gray-blood PSIR and free-breathing 3D CMRA.

**Results:**

GB-BOOST was successfully performed on all 23 patients and was able to efficiently acquire intrinsically co-registered 3D PSIR and CMRA images with 1.2 mm^3^ resolution in 9.4 ± 1.3 min. Compared with 2D gray-blood PSIR, 3D PSIR GB-BOOST had comparable scar area detection performance without significant differences in image contrast of scar-to-blood (0.42 ± 0.40 vs. 0.30 ± 0.43, p = 0.38), scar-to-myocardium (1.09 ± 0.27 vs. 1.02 ± 0.32, p = 0.30), and blood-to-myocardium (0.67 ± 0.19 vs. 0.72 ± 0.23, p = 0.56). Compared with single-contrast 3D CMRA sequence, 3D T2prep GB-BOOST showed comparable image quality and quantitative vessel metrics of coronary arteries.

**Conclusion:**

The proposed GB-BOOST sequence can achieve simultaneous co-registered 3D whole-heart gray-blood PSIR and CMRA in a single scan with image contrast and image quality comparable with separately acquired images.

## Graphical abstract

**Figure:**
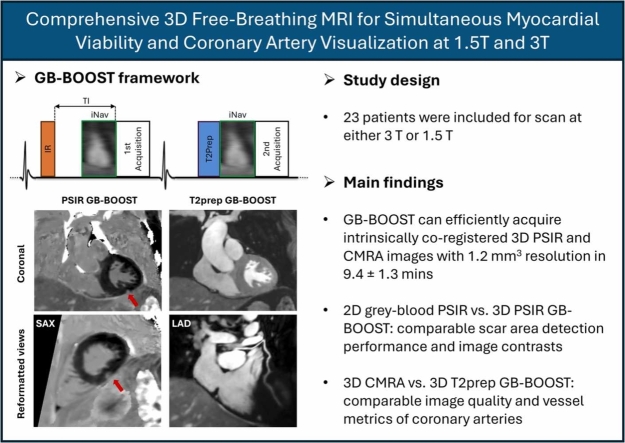

## 1. Background

Cardiovascular magnetic resonance (CMR) imaging has been widely used for the non-invasive assessment of various cardiac diseases with the ability to provide multi-contrast images [1], [2], [3]. Coronary magnetic resonance angiography (CMRA) has the potential to enable radiation-free visualization of the complex anatomy of the coronary arteries [4]. CMRA is usually performed with bright-blood contrast using a T2 preparation (T2prep) pulse to improve blood-to-myocardium contrast [5]. In concert with free-breathing 3D high-resolution imaging techniques, CMRA has been shown to provide good performance, comparable to computed tomography for proximal and mid-vessel segments [6], [7].

On the other hand, late gadolinium enhancement (LGE) is the preferred technique for detection of myocardial scar [8]. Conventional 2D LGE is performed with inversion recovery (IR) or phase-sensitive inversion recovery (PSIR) sequences, which achieve bright-blood contrast by nulling the signal of normal myocardium [9]. Although bright-blood LGE has good scar-to-myocardium contrast, it exhibits a poor scar-to-blood contrast [10]. To optimize the image contrast of LGE, different techniques have been proposed such as black-blood and gray-blood (also known as dark-blood) sequences. These sequences can suppress the signal of blood and demonstrate better results in scar depiction than bright-blood LGE, especially for subendocardial and papillary muscle myocardial infarctions [11], [12], [13], [14], [15], [16]. Some of these techniques combine additional preparation pulses with IR, such as T2prep or magnetization transfer [11], [12], [13], [14], [17]. However, these pulses incorporate additional T2 or magnetization transfer weighting to the T1-weighted LGE images, potentially decreasing the scar signal [18]. For example, scar signal with T2prep-IR preparation will be attenuated in cases of edema that also has T2 elevation, which is often seen in acute myocardial infarction or acute myocarditis [18]. Alternatively, a gray-blood PSIR LGE sequence without additional preparation pulses has previously been proposed by nulling the signal of the blood pool using IR preparation only, which can maintain T1 weighting while increasing the scar-to-blood contrast [15], [16], [19].

Multi-contrast CMR with LGE and CMRA imaging is promising for the combined evaluation of tissue characteristics and cardiac and coronary anatomy, especially for coronary artery disease (CAD). However, in clinical practice, LGE and CMRA images are typically acquired separately using 2D and 3D sequences, respectively. As a result, the mismatch in spatial resolution and slice positioning between the two modalities limits their utility for accurate image fusion and for reliably assigning myocardial scars to the corresponding coronary artery territories [20]. Therefore, a protocol with joint 3D whole-heart LGE and CMRA imaging is of high clinical interest.

In this study, we sought to achieve a one-stop imaging technique that integrates 3D gray-blood PSIR LGE and 3D CMRA by proposing a novel, free-breathing whole-heart simultaneous Gray-Blood and Bright-blOOd phase SensiTive inversion recovery (GB-BOOST) sequence. GB-BOOST consists of a 2-heartbeat sequence scheme similar to a previous gray-blood PSIR sequence that acquires two interleaved 3D volumes, i.e., an IR volume and a phase reference volume [19]. However, GB-BOOST uses an additional T2prep for the reference volume to induce a bright-blood contrast for CMRA while maintaining polarity of the reference phase. Thus, compared with gray-blood PSIR LGE, GB-BOOST can efficiently acquire two contrast-weighted images without increasing the total scan time. In-vivo validation experiments included 23 patients and were performed on either a 3T or a 1.5T clinical scanner. The performance of the proposed sequence was compared with clinical 2D gray-blood PSIR LGE and 3D CMRA, respectively.

## 2. Methods

### 2.1. GB-BOOST sequence framework

**Fig. 1:**
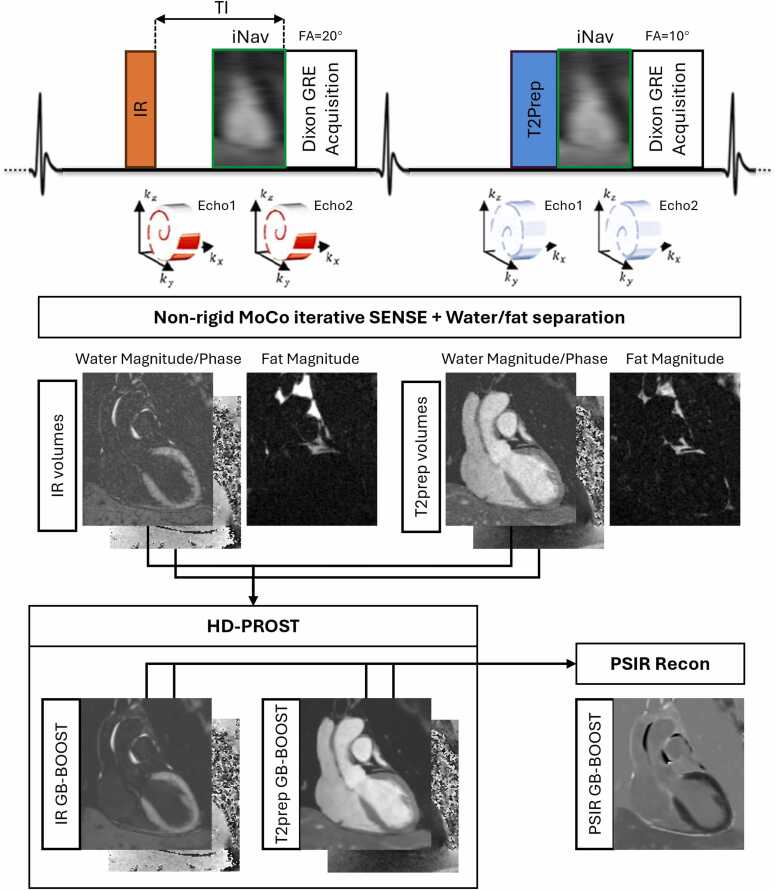
Sequence and reconstruction framework of the proposed GB-BOOST sequence. Two interleaved electrocardiogram-triggered 3D volumes are acquired using 2-point Dixon GRE acquisition with IR and T2prep, respectively. iNAV enables 100% respiratory scan efficiency. A variable-density Cartesian trajectory with spiral-like profile order and golden-angle step is adopted with five-fold undersampling. Inline non-rigid MoCo iterative SENSE reconstruction and water/fat separation are performed to obtain IR and T2prep water/fat volumes, which is followed by offline HD-PROST denoising and PSIR reconstruction to finally produce IR GB-BOOST, T2prep GB-BOOST, and PSIR GB-BOOST images. *GRE* gradient echo, *IR* inversion recovery, *T2prep* T2 preparation, *iNAV* image navigator, *MoCo* motion corrected, *HD-PROST* high-dimensional patch-based low-rank regularization, *PSIR* phase sensitive inversion recovery, *GB-BOOST* Gray-Blood and Bright-blOOd phase SensiTive inversion recovery

The proposed GB-BOOST research sequence acquires two interleaved electrocardiogram-triggered 3D volumes in a 2-heartbeat scheme. The acquisitions in odd and even heartbeats are prepared with IR and T2prep pulses, respectively (Fig. 1). The IR-prepared volume is for gray-blood LGE, and the inversion recovery time (TI) is set to null blood signal according to a subject-specific Bloch equation simulation using tissue T1 values measured by a scout post-contrast T1 map. A demonstrative simulation tool for the TI selection is available online at https://dongyuesi-kcl.github.io/TI_signal-calc/. The second volume provides a phase reference for the phase-sensitive reconstruction of the LGE volume, enabling the gray-blood contrast generation. In addition, the T2prep in the second interleave allows for improved contrast between blood pool and myocardium while maintaining polarity of the phase. Thus, the phase reference volume can also be used for CMRA imaging, thereby increasing imaging efficiency and allowing to relate epicardial CAD to the affected coronary territory (LGE). A two-dimensional image navigator (iNAV) is acquired before the acquisition of each volume to detect respiratory motion for motion-corrected reconstruction, enabling free-breathing acquisition with 100% respiratory scan efficiency [21]. An undersampled variable-density Cartesian trajectory with spiral-like profile order and golden-angle step (VD-CASPR) is adopted [22].

The GB-BOOST framework is compatible with different readout sequences for the application at different field strengths. For imaging at higher field strengths such as 3T, 2-point bipolar Dixon gradient echo (GRE) acquisition is performed with flip angles of 20° and 10° for the two volumes, respectively (Fig. 1). Inline non-rigid motion-corrected iterative SENSE reconstruction and water/fat separation is performed to generate IR and T2prep water and fat volumes [23]. As the signal polarity is lost after water/fat separation [19], the phase images of the water volumes inherit those of the first echo volumes. Offline high-dimensional patch-based low-rank regularization (HD-PROST) denoising is performed on the complex-valued water volumes to produce IR GB-BOOST LGE and T2prep GB-BOOST CMRA images [24], which are then used to generate a PSIR GB-BOOST LGE image with a PSIR reconstruction [19]. For the GB-BOOST sequence with bSSFP acquisition (Fig. S1), non-rigid motion-corrected iterative SENSE and PSIR reconstruction are both performed inline, which can directly produce three sets of volumes (IR, T2prep, and PSIR). Then, offline HD-PROST denoising is performed on the T2prep and PSIR volumes to finally generate T2prep GB-BOOST CMRA and PSIR GB-BOOST LGE images.

### 2.2. Simulation experiments

To investigate the signal behavior of the two volumes of GB-BOOST using IR and T2prep preparation, respectively, simulation experiments were conducted comparing the signal of GB-BOOST with that acquired by single-contrast PSIR and CMRA reference sequences. In the simulation, T1/T2 values of normal myocardium, blood, and scar were 600/40 ms, 400/150 ms, and 300/50 ms, respectively. GB-BOOST with Dixon GRE acquisition scheme was simulated with a TR of 5.23 ms, 18 lines per readout, flip angles of 20/10°, and a T2prep duration of 50 ms. According to the simulation of the signal evolution during multiple heartbeats, the sequence would reach a steady state after three repetitions, presenting the same signal intensity in repeated units of the two heartbeats. To achieve gray-blood contrast in the odd heartbeat, TI was selected to null the blood signal according to the simulation-based scout method using the Bloch equation, given the blood T1/T2 values and acquisition parameters [11], [25]. Then, the signal intensities of different tissues in both the odd and even heartbeats were calculated. The simulation was performed with simulated heart rates ranging from 40 to 100 bpm, with a step size of 10 bpm. As references, the signals of conventional gray-blood PSIR and bright-blood CMRA were also simulated at different heart rates. For PSIR, the signal intensity was simulated using the same imaging parameters as in GB-BOOST. TI was also selected to null blood signal, but no T2prep was used in the even heartbeats. For T2prep CMRA, only one volume was simulated using a T2prep with 50 ms duration and a flip angle of 20°. Numerical simulations were implemented in MATLAB R2023a (MathWorks, Natick, Massachusetts).

### 2.3. In-vivo experiments

Totally 23 clinical cardiac patients (11 males, 58 ± 16 years) were recruited for additional research scans using the proposed GB-BOOST sequence. The human experiments were approved by the local institutional review board. Written informed consent was obtained from all subjects before imaging. As the patients were scheduled according to clinical arrangements, all in-vivo experiments were randomly performed on either a 3T MR scanner (MAGNETOM Vida, Siemens Healthineers, Erlangen, Germany) or a 1.5 T MR scanner (MAGNETOM Sola, Siemens Healthineers, Erlangen, Germany) each equipped with an 18-channel chest coil and a 16-channel spine coil.

Clinical breath-held 2D bright-blood PSIR and 2D gray-blood PSIR, and research free-breathing 3D GB-BOOST and 3D CMRA were sequentially scanned at approximately 10–20 mins after the injection of a gadolinium-based contrast agent (GADOVIST 1.0 (gadobutrol), Bayer Healthcare, Berlin, Germany) at a dose of 0.15 mmol/kg. Clinical 2D PSIR sequences were acquired in multiple long-axis and short-axis views covering the left ventricle with in-plane resolution = 1.4 mm^2^ and slice thickness = 8 mm. Bright-blood and gray-blood contrasts were achieved by setting TI to null the signal of normal myocardium and blood pool, respectively. TI scout imaging was performed with a 2-heartbeat Look-Locker sequence. Both 3D GB-BOOST and 3D CMRA sequences were performed after 2D PSIR imaging in coronal orientation with isotropic spatial resolution of 1.2 mm^3^ covering the whole heart with field-of-view (FOV) = 340 × 340 × ∼100 mm^3^. For 3T imaging, Dixon GRE readout was used for the two 3D sequences, with TR/TE1/TE2 = 5.23/1.67/3.18 ms, bandwidth = 827 Hz/pixel, T2prep duration = 50 ms, iNAV with 14 echoes and 3° flip angle, and VD-CASPR with 5-fold undersampling. 3D CMRA had a similar sequence scheme as GB-BOOST including the use of iNAV, but only one contrast with T2prep preparation was acquired using a flip angle of 20°. While for 1.5T imaging, bSSFP readout was used for the two 3D sequences, with flip angle = 90°, TR/TE = 3.33/1.46 ms, bandwidth = 967 Hz/pixel, T2prep duration = 40 ms, iNAV with 14 echoes and a linear ramp, and VD-CASPR with 4-fold undersampling. Before performing GB-BOOST, scout post-contrast T1 map was acquired with modified Look-Locker inversion recovery 4(1)3(1)2 sequence [26], with FOV = 307 × 360 mm^2^, resolution = 1.4 mm^2^, and slice thickness = 8 mm. A middle short-axis slice was imaged, and a circular region of interest (ROI) within the blood pool was drawn inline on the scanner to estimate the average blood T1 for the signal simulation to select TI.

### 2.4. Data analysis

To evaluate the performance of GB-BOOST for LGE imaging, scar detection analysis was performed on 3D PSIR GB-BOOST LGE images, and the results were compared with those of 2D gray-blood PSIR. The assessment was performed by an experienced reviewer (7 years of experience in CMR). The 3D volumes were manually reformatted to short-axis views with spatial orientations matching the related 2D images before data processing. About 80 short-axis slices with 1.2 mm isotropic resolution were generated for each 3D volume. Scar detection analysis was carried out on each dataset using the 17-segment American Heart Association (AHA) model [27]. Specifically, all short-axis slices covering the left ventricle were divided into 17 AHA segments, and each segment was visually inspected to identify scar [19]. 2D and 3D images were analyzed without side-by-side comparison between each other on a per-patient basis. Furthermore, for those patients with positive LGE findings, the contrasts between scar, blood, and normal myocardium were calculated for both 2D gray-blood PSIR and 3D PSIR GB-BOOST LGE. A representative short-axis slice that showed scar was selected for each data, then, ROIs of scar, blood, and myocardium were manually drawn to measure the average signal intensity (S) of each tissue, respectively. Given two tissues, A and B, selected from scar, blood, and myocardium, the A-to-B contrast is defined as:

$${\mathrm{Contrast}}_{A/B}=\frac{S_{A}-S_{B}}{\sqrt{S_{\mathrm{Scar}}^{2}+S_{\mathrm{Blood}}^{2}+S_{\mathrm{Myo}}^{2}}}$$

where S_A_ is the signal intensity of tissue A and S_B_ is the signal intensity of tissue B. The contrast of scar-to-blood, scar-to-myocardium, and blood-to-myocardium were calculated, and the statistical differences of tissue contrasts measured by different sequences were analyzed using paired two-tailed Student's t-test (α = 0.05).

Additional analysis was also performed to measure the scar mass for both 2D gray-blood PSIR and 3D PSIR GB-BOOST images using CVI42 software (Circle Cardiovascular Imaging, Calgary, Alberta, Canada). The whole left ventricle was manually segmented on each 2D or 3D dataset. Scar mass quantification was performed by manually segmenting the scar based on signal intensity for each scarred patient. The scar mass was expressed as percentage of total left-ventricular mass. Bland-Altman analysis was performed to compare scar mass measured by 2D and 3D sequences.

To evaluate the performance of GB-BOOST for CMRA imaging, quantitative coronary artery metrics were analyzed on 3D T2prep GB-BOOST CMRA images, and the results were compared with those of 3D CMRA with matched spatial resolution and location. Vessel sharpness (first 4 cm) and maximum visible vessel length of left anterior descending artery (LAD), left circumflex artery (LCx), and right coronary artery (RCA) were measured using the “Soap-Bubble” software [28]. Paired two-tailed Student's t-test (α = 0.05) was used to analyze the statistical differences of vessel metrics measured by different sequences.

## 3. Results

### 3.1. Simulation

Simulated signal intensities of normal myocardium, blood pool, and scar of IR and T2prep volumes acquired with different sequences are shown in Fig. S2. For the IR volume with gray-blood contrast, both PSIR and GB-PSIR successfully null the signal of the blood pool using the proposed simulation-based TI scout method. At the same time, the signal intensity of scar was maintained, producing an excellent contrast between the scar and blood pool. Although the signal intensity of myocardium in GB-BOOST was slightly increased compared to that in conventional PSIR, especially for higher heart rate, it was still lower than the blood signal and could achieve a gray-blood contrast (Fig. S2A). For the T2prep volume, both the signal intensities of blood and myocardium in GB-BOOST are comparable with those in CMRA (Fig. S2B), indicating negligible influence from the interleaved acquisition of the other volume with IR preparation.

### 3.2. In-vivo experiments

In-vivo experiments of the proposed 3D GB-BOOST sequence were successfully performed on all 23 patients in an average scan time of 9.4 ± 1.3 min with an average heart rate of 67 ± 13 bpm. Twelve patients were scanned at 3T (9.6 ± 1.5 min), and 11 were scanned at 1.5T (9.1 ± 1.0 min). Ten patients (5 males, 52 ± 15 years) also underwent the 3D CMRA scan, which had an average scan time of 4.8 ± 0.3 min with an average heart rate of 67 ± 14 bpm. The patient characteristics and scan times are summarized in Table 1. Among all the patients, seven demonstrated positive LGE findings.

**Table 1:** Patient characteristics and scan times

Patient #	Gender	Age (y)	HR (bpm)	Field strength (Tesla)	3D GB-BOOST scan time (min)	3D CMRA scan time (min)	Positive LGE
1	F	63	67	3	9.5	4.6	No
2	F	46	63	3	8.5	4.2	No
3	F	26	44	3	13.5	4.9	No
4	M	36	53	3	10.5	5.2	No
5	F	59	54	3	10.2	5.0	Yes
6	M	60	79	3	9.0	4.6	No
7	M	60	94	3	9.5	4.7	No
8	F	60	56	3	7.2	N/A	Yes
9	F	72	71	3	9.0	N/A	Yes
10	M	59	63	3	10.9	N/A	No
11	M	74	63	3	8.3	N/A	No
12	F	73	40	3	9.0	N/A	Yes
13	M	74	75	1.5	9.3	4.6	Yes
14	F	66	68	1.5	10.2	5.1	No
15	M	34	70	1.5	9.9	4.8	No
16	M	80	58	1.5	10.4	N/A	Yes
17	F	77	71	1.5	9.2	N/A	No
18	F	76	81	1.5	8.5	N/A	No
19	F	53	73	1.5	8.3	N/A	No
20	F	37	89	1.5	6.8	N/A	No
21	M	67	78	1.5	9.5	N/A	Yes
22	M	60	55	1.5	9.4	N/A	No
23	M	23	72	1.5	8.5	N/A	No
Mean±SD	-	58 ± 16	67 ± 13	-	9.4±1.3	4.8±0.3	

*HR* heart rate, *bpm* beats per minute, *N/A* not applicable

**Fig. 2:**
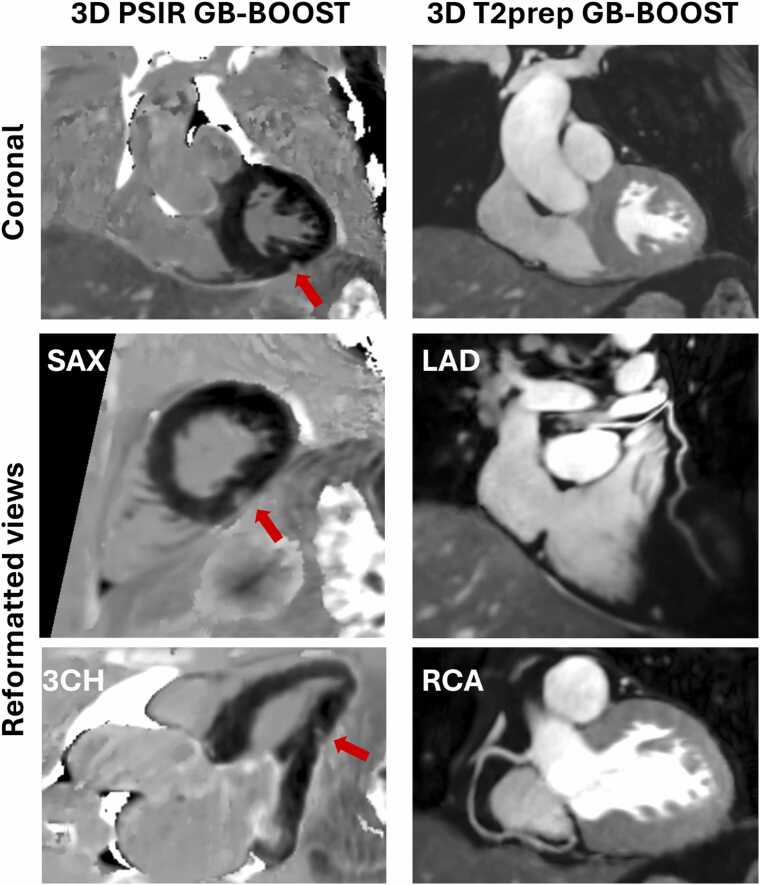
Representative images of a 59-year-old female patient (#5) acquired with the proposed 3D GB-BOOST sequence at 3T. PSIR and T2prep GB-BOOST images were simultaneously acquired and had intrinsically co-registered spatial location. 3D whole-heart imaging with 1.2 mm isotropic resolution enables reformatted views in different orientations. PSIR GB-BOOST was reformatted to SAX and 3CH views, while T2prep GB-BOOST performed curved planar reconstruction to show LAD and RCA, respectively. PSIR GB-BOOST shows multiple subtle scar areas across the entire heart, which are indicated with red arrows. *3D* three-dimensional, *GB-BOOST* Gray-Blood and Bright-blOOd phase SensiTive inversion recovery, *PSIR* phase sensitive inversion recovery, *T2prep* T2 preparation, *SAX* short-axis, *3CH* 3-chamber, *LAD* left anterior descending artery, *RCA* right coronary artery

Representative images of patient #5 scanned at 3T are shown in Fig. 2. PSIR GB-BOOST provided gray-blood LGE images, showing a good contrast for the detection of myocardial scar, which is consistent with 2D PSIR LGE findings (Fig. S3). T2prep GB-BOOST presented a bright-blood contrast for CMRA. According to the reformatted curved planar reconstruction, both LAD and RCA can be clearly visualized.

**Fig. 3:**
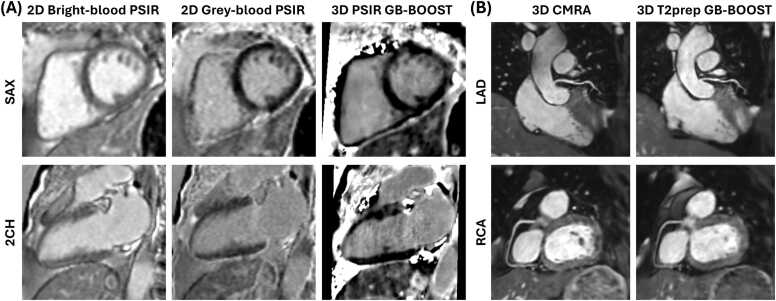
Representative images of a 66-year-old female patient (#14) acquired with different 2D and 3D sequences at 1.5T. (**A**) LGE images acquired with 2D bright-blood PSIR, 2D gray-blood PSIR, and 3D PSIR GB-BOOST in SAX and 2CH views. The results showed negative LGE findings. 3D PSIR GB-BOOST had image contrast comparable with 2D gray-blood PSIR by nulling the signal of blood pool. (**B**) CMRA images acquired with 3D CMRA and 3D T2prep GB-BOOST with curved planar reconstruction to show LAD and RCA respectively. 3D T2prep GB-BOOST showed image contrast comparable with 3D CMRA, presenting good image quality for the depiction of the structure of both LAD and RCA. *2D* two-dimensional, *3D* three-dimensional, *LGE* late gadolinium enhancement, *PSIR* phase sensitive inversion recovery, *GB-BOOST* Gray-Blood and Bright-blOOd phase SensiTive inversion recovery, *SAX* short-axis, *2CH* 2-chamber, *CMRA* coronary magnetic resonance angiography, *T2prep* T2 preparation, *LAD* left anterior descending artery, *RCA* right coronary artery

Representative images of patient # 14 acquired with 2D PSIR sequences, 3D GB-BOOST, and 3D CMRA at 1.5T are shown in Fig. 3. 3D PSIR GB-BOOST LGE had image contrast comparable with 2D gray-blood PSIR by nulling the signal of the blood pool. In addition, visual comparison indicates that 3D T2prep GB-BOOST CMRA showed image contrast comparable with 3D CMRA, presenting good image quality for the depiction of the course and structure of both LAD and RCA.

**Fig. 4:**
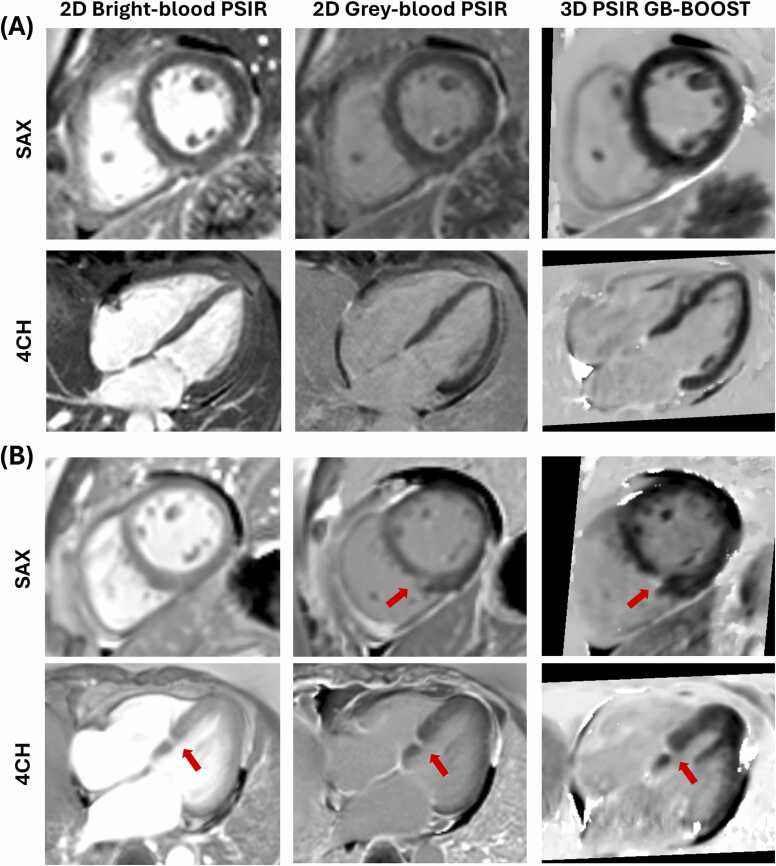
Representative LGE images of other two patients acquired with 2D bright-blood PSIR, 2D gray-blood PSIR, and 3D PSIR GB-BOOST at 3T in SAX and 4CH views. (**A**) Patient # 1 with negative LGE findings. (**B**) Patient #9 with a small scar area in the inferior-septal segment indicated by red arrows. Both 2D gray-blood PSIR and 3D PSIR GB-BOOST captured the related SAX slice presenting scar, while 2D bright-blood PSIR missed the related SAX slice. The scar was best captured in the 4CH slices. GB-BOOST with 3D whole-heart coverage allowed scar detection both in the reformatted SAX and 4CH views. *LGE* late gadolinium enhancement, *2D* two-dimensional, *PSIR* phase sensitive inversion recovery, *3D* three-dimensional, *GB-BOOST* Gray-Blood and Bright-blOOd phase SensiTive inversion recovery, *SAX* short-axis, *4CH* 4-chamber

For the comparison of 2D and 3D LGE images, representative images for two other patients (# 1 and # 9) scanned at 3T are shown in Fig. 4. The results show that 3D PSIR GB-BOOST LGE had image contrast comparable to 2D gray-blood PSIR and better contrast than 2D bright-blood PSIR. Patient # 9 presented a small scar area in the inferior-septal segment, which was best captured in the 4-chamber LGE images acquired with either 2D or reformatted 3D sequences.

**Fig. 5:**
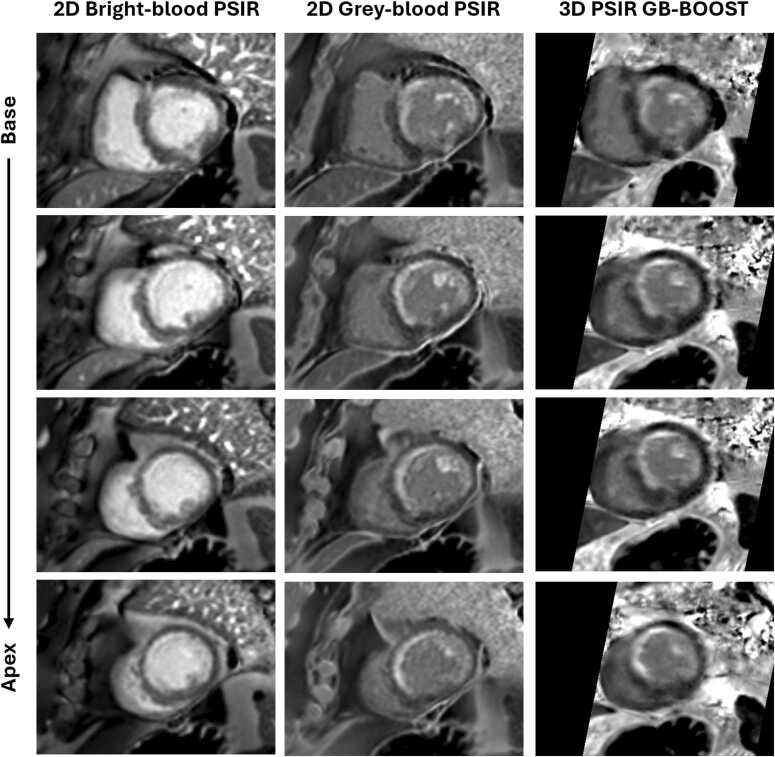
LGE images of a 74-year-old male patient (#13) acquired with 2D bright-blood PSIR, 2D gray-blood PSIR and 3D PSIR GB-BOOST at 1.5T. Four short-axis slices from base to apex are shown. There was a large area of subendocardial myocardial infarction in the left anterior descending artery and left circumflex territory. Bright-blood PSIR presented a poor contrast especially for the detection of subendocardial and papillary muscle enhancement, while 3D PSIR GB-BOOST and 2D gray-blood PSIR had superior contrast, presenting consistent findings regarding the scar area across the heart. *LGE* late gadolinium enhancement, *2D* two-dimensional, *PSIR* phase sensitive inversion recovery, *3D* three-dimensional, *GB-BOOST* Gray-Blood and Bright-blOOd phase SensiTive inversion recovery, *T2prep* T2 preparation

**Fig. 6:**
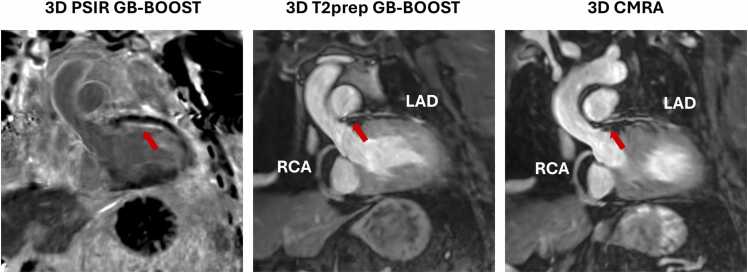
Curved planar reconstructions of 3D GB-BOOST and 3D CMRA of the same patient (#13) shown in Fig. 5. The left main coronary artery occlusion treated with PCI was confirmed by the 3D T2prep GB-BOOST and 3D CMRA images. Both sequences demonstrated a signal void around the left main stem due to the PCI stents (red arrows), which was not seen in the RCA. In addition, the PSIR GB-BOOST image with the same curved planar reconstruction showed scar area highly correlated with the left main coronary artery territory (red arrow). *PSIR* phase sensitive inversion recovery, *3D* three-dimensional, *GB-BOOST* Gray-Blood and Bright-blOOd phase SensiTive inversion recovery, *RCA* right coronary artery, *PCI* percutaneous coronary intervention, *CMRA* coronary magnetic resonance angiography, *T2prep* T2 preparation

Fig. 5 shows the LGE images of patient # 13 acquired at 1.5T. This patient had known left-ventricular impairment secondary to CAD with chronic total occlusion of the left main coronary artery. The LAD and the LCx were being supplied from collaterals from the RCA. The patient subsequently underwent a percutaneous coronary intervention (PCI) to the left main coronary artery, and CMR scan was performed one year after the treatment. According to the LGE images, there was an area of subendocardial myocardial infarction in the LAD and LCx territory. The 2D bright-blood PSIR images provided poor contrast especially for the detection of subendocardial and papillary muscle enhancement, while the 3D PSIR GB-BOOST LGE and 2D gray-blood PSIR images provided superior contrast, presenting consistent findings regarding scar location and burden throughout the whole heart. Curved planar reconstructions of the 3D GB-BOOST and 3D CMRA of the same patient are shown in Fig. 6. The left main coronary artery occlusion treated with PCI was confirmed by the 3D T2prep GB-BOOST CMRA and 3D CMRA images. In addition, the PSIR GB-BOOST LGE image, post-processed with the same curved planar reconstruction, showed the scar area, which was strongly correlated with the LAD territory.

### 3.3. Quantitative analysis

**Fig. 7:**
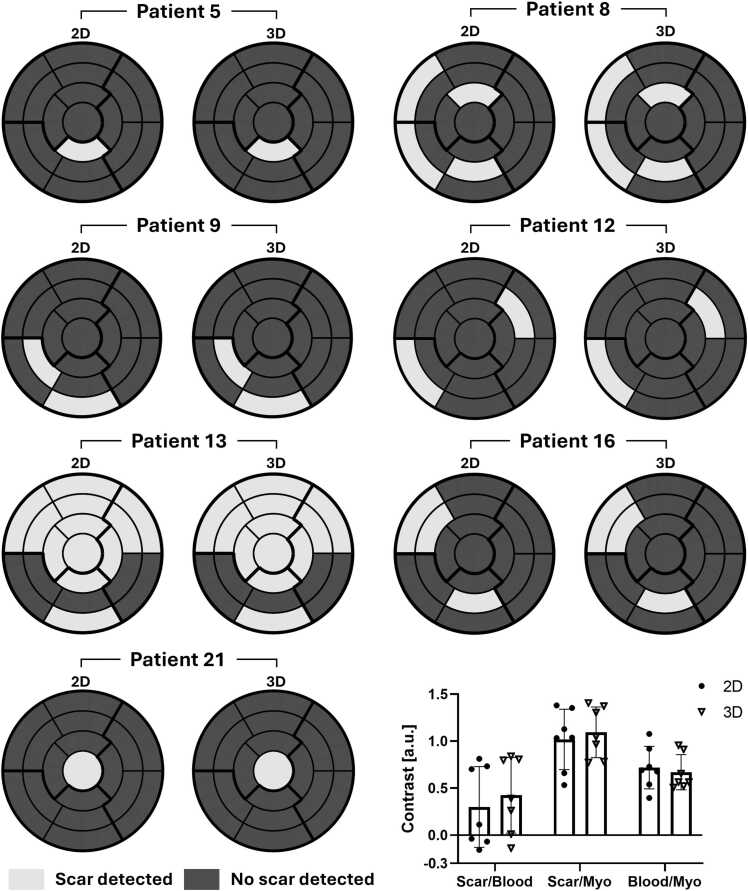
Scar detection analysis results with 2D gray-blood PSIR and 3D PSIR GB-BOOST of the 7 patients presenting positive LGE findings. Scar area detection in 17-segment AHA model is shown in bull’s eye plot for each patient. Image contrast between different tissues, including scar-to-blood (Scar/Blood), scar-to-myocardium (Scar/Myo), and blood-to-myocardium (Blood/Myo) are shown. No significant differences were found in the contrast of Scar/Blood (0.42 ± 0.40 vs. 0.30 ± 0.43, p = 0.38), Scar/Myo (1.09 ± 0.27 vs. 1.02 ± 0.32, p = 0.30), and Blood/Myo (0.67 ± 0.19 vs. 0.72 ± 0.23, p = 0.56) between 2D and 3D results. *2D* two-dimensional, *PSIR* phase sensitive inversion recovery, *3D* three-dimensional, *GB-BOOST* Gray-Blood and Bright-blOOd phase SensiTive inversion recovery, *LGE* late gadolinium enhancement, *AHA* American Heart Association

Scar detection analysis with the 17-segment AHA model was performed in the 7 patients presenting positive LGE findings (Fig. 7). No enhancement was found for the other patients according to either 2D or 3D LGE images. Excellent agreement in scar detection and scar location across the whole-heart was obtained between the 2D gray-blood PSIR and 3D PSIR GB-BOOST LGE sequences for those 7 patients. When comparing image contrast between 3D and 2D gray-blood LGE images for the 7 patients, no significant differences were found in the contrast of scar-to-blood (0.42 ± 0.40 vs. 0.30 ± 0.43, p = 0.38), scar-to-myocardium (1.09 ± 0.27 vs. 1.02 ± 0.32, p = 0.30), and blood-to-myocardium (0.67 ± 0.19 vs. 0.72 ± 0.23, p = 0.56). The scar mass measured by 2D and 3D LGE images also showed a good agreement (Fig. S4).

**Fig. 8:**
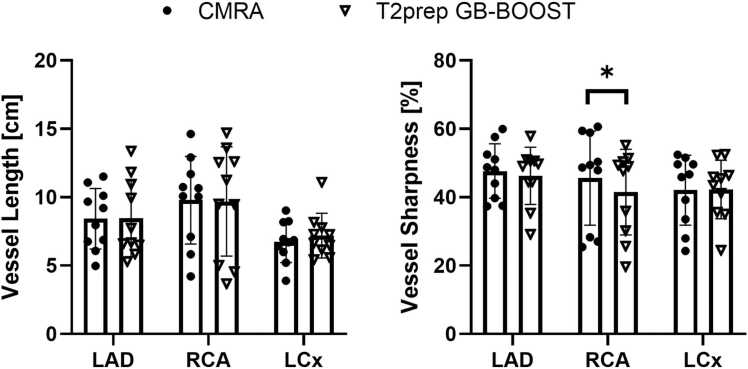
Vessel length and vessel sharpness of LAD, RCA, and LCx measured with 3D CMRA and 3D T2prep GB-BOOST of the 10 patients scanned by both sequences. *Indicates statistical significance (p = 0.03). *LAD* left anterior descending artery, *RCA* right coronary artery, *LCx* left circumflex coronary artery, *3D* three-dimensional, *CMRA* coronary magnetic resonance angiography, *T2prep* T2 preparation, *GB-BOOST* Gray-Blood and Bright-blOOd phase SensiTive inversion recovery

Quantitative coronary artery metrics of the 10 patients scanned with both the 3D T2prep GB-BOOST CMRA and 3D CMRA sequence are shown in Fig. 8. No significant differences were found between the 3D T2prep GB-BOOST CMRA and 3D CMRA regarding vessel length of all arteries including LAD (8.5 ± 2.8 vs. 8.4 ± 2.2 cm, p = 0.92), RCA (9.7 ± 4.0 vs. 9.8 ± 3.2 cm, p = 0.83) and LCx (7.2 ± 1.6 vs. 6.7 ± 1.5 cm, p = 0.36), and vessel sharpness of both LAD (46.3 ± 8.4 vs. 47.7 ± 8.0%, p = 0.40) and LCx (42.3 ± 8.6 vs. 42.1 ± 10.2%, p = 0.92). While the T2prep GB-BOOST CMRA presented a slightly lower vessel sharpness for the RCA compared to 3D CMRA (41.5 ± 12.5 vs. 45.6 ± 13.7%, p = 0.03).

## 4. Discussion

In this proof of concept study, we proposed GB-BOOST, a novel whole-heart sequence that provides two intrinsically co-registered 3D high-resolution volumes with IR and T2 preparation, respectively, enabling simultaneous 3D gray-blood LGE and CMRA in a single free-breathing scan. 2D iNAVs were used to enable 100% respiratory scan efficiency and non-rigid motion-compensated image reconstruction. Preliminary validation experiments were successfully performed on 23 clinical cardiac patients using both 1.5T and 3T scanners. The proposed GB-BOOST sequence was able to achieve whole-heart coverage with an isotropic resolution of 1.2 mm in an efficient scan time of about 10 min. Compared with 2D gray-blood PSIR, 3D PSIR GB-BOOST LGE demonstrated comparable image contrast and scar area detection performance. Compared with a single-contrast 3D CMRA sequence, 3D T2prep GB-BOOST CMRA provided comparable image quality and vessel metrics.

Conventional LGE imaging with IR preparation and bright-blood contrast is the reference standard for non-invasive assessment of myocardial viability because of the good contrast between myocardial scar and viable myocardium. However, suboptimal endocardial scar-to-blood contrast can influence the accurate delineation of the border between scar and the adjacent blood pool. IR preparation based gray-blood LGE was proposed to improve endocardial scar-to-blood contrast by nulling the signal of the blood pool without the need for additional preparation pulses [16]. However, as the signal from the myocardium has negative phase in the first (odd) heartbeat, PSIR reconstruction using a phase reference image is necessary to recover the signal polarity. Thus, gray-blood LGE was previously implemented employing a 2-heartbeat PSIR-like sequence scheme with either 2D or 3D imaging [15], [19]. The acquisition of an additional reference image however doubles the total scan time and thus reduces the scan efficiency, especially for 3D imaging. Here, 3D GB-BOOST adopted a similar 2-heartbeat acquisition scheme as the initially proposed 3D gray-blood PSIR technique [19]. However, GB-BOOST includes an additional T2prep pulse in the second (even) heartbeat required for the phase reference image to induce a bright-blood contrast. Thus, the reference image can also be used to reconstruct a CMRA dataset. Compared with conventional PSIR LGE, GB-BOOST takes full advantage of the reference image, producing an additional bright-blood CMRA image along with the gray-blood LGE image without increasing the total scan time.

As GB-BOOST incorporates an additional T2prep pulse in the even heartbeats, the signal behavior during data acquisition is different from conventional PSIR sequence that only employs IR pulses. Thus, the TI delay for nulling the blood signal in GB-BOOST can be different from that in conventional PSIR. In this study, a 2-heartbeat Look-Locker scan was used as a TI scout for the conventional PSIR sequence, but was not compatible with GB-BOOST. As an alternative, we adopted a Bloch equation simulation method using blood T1, patient heart rate, and sequence acquisition parameters to select the correct TI for blood nulling. The simulation-based TI scout is a well-established method that has been used in previous studies for dark-blood LGE imaging [11], [25]. The optimal TI can be calculated easily given the above parameters, avoiding manual selection of the nulling point based on the radiographer’s experiences. According to the simulation (Fig. S2A) and in-vivo results of the seven patients with positive LGE findings, GB-BOOST achieved good image contrasts between scar, blood and myocardium, which did not show any significant differences compared with those of the 2D gray-blood PSIR sequence (Fig. 7). Therefore, the simulation-based scout can estimate an accurate TI delay time for GB-BOOST, thus maintaining a good gray-blood contrast for the PSIR LGE images despite the addition of a T2prep prepulse in even heartbeats.

Compared to clinical 2D PSIR imaging, PSIR GB-BOOST also has the advantage of larger spatial coverage and higher spatial resolution due to the nature of 3D free-breathing imaging. In clinical practice, 2D LGE imaging is usually performed in 10–15 short-axis slices covering the left ventricle and several long-axis slices, such as 2-, 3-, and 4-chamber views [20]. Due to the limited number of slice positions, large slice thickness, and potential gaps between slices, 2D LGE carries the risk of false negatives, as the slice containing scar could be missed, especially for small lesions such as in patient #9 (Fig. 4). During the exam, additional 2D slices are often required with customized adjustments to slice position and orientation to better delineate scarred regions [29]. However, this workflow adds complexity for the radiographer and prolongs overall scan time. In contrast, 3D imaging with GB-BOOST enables whole-heart coverage with isotropic high resolution (1.2 mm) and no slice gaps, allowing multiplanar reformatting in any arbitrary orientation or standard cardiac views. This comprehensive visualization reduces the risk of missing critical findings, such as small areas of myocardial scar.

The reference 3D CMRA image was acquired with a T2prep sequence using the same acquisition parameters as GB-BOOST. Comparable performance was observed regarding image contrast (Fig. S2B) and depiction of both LAD and RCA arteries (Fig. 8) between T2prep GB-BOOST and the reference CMRA sequence. Statistical analysis showed that T2prep GB-BOOST resulted in a slightly lower vessel sharpness for the RCA compared to CMRA (41.5 ± 12.5 vs. 45.6 ± 13.7%, p = 0.03). This may be because GB-BOOST had a relatively longer total scan time than the conventional CMRA sequence. As both 3D sequences were performed at the end of the clinical scan, patients may have been already tired, especially after a long CMR session that required multiple breath-holds. Thus, GB-BOOST may suffer more from image blurring due to the potential irregular respiratory motion and heart rate variation during the free-breathing scan period. Imaging at an earlier time could potentially minimize this effect.

In summary, GB-BOOST can efficiently provide gray-blood PSIR LGE and CMRA images/volumes with good image quality comparable with separately acquired conventional 2D LGE and 3D CMRA images. Moreover, the two datasets are intrinsically co-registered, benefiting the joint evaluation of cardiac structure and myocardial viability, such as shown in one CAD patient (Fig. 6). Combining the information of both coronary artery anatomy/integrity and myocardial tissue characterization for the detection of CAD could potentially help us to better understand the relationship between epicardial CAD and its effect on the downstream myocardium and thus improve prognosis as both the area of myocardial scar and the degree of obstructive luminal disease are associated with adverse cardiac events [30], [31]. Although promising results have been demonstrated, this study is limited by the relatively small number of patients with positive LGE findings. Validation experiments with larger cohort of patients, especially those with CAD are thus required in the future.

## 5. Limitations

In this study, 3D GB-BOOST sequence was always acquired after the clinical 2D sequences without random scan ordering. Due to contrast washout and changes in T1 during the scan and after contrast injection, image contrast between different tissues may vary. However, as GB-BOOST was performed immediately after 2D GB-PSIR, this effect had been reduced to the minimum. In addition, with accurate TI selection based on simulation, GB-BOOST should maintain a good gray-blood contrast, comparable to that of the 2D sequence acquired earlier. Nevertheless, further comparison between 2D and 3D sequences using a randomized scan order is warranted in future studies.

In addition, the experiments were performed on either a 1.5T or a 3T scanner according to clinical schedule. Since this was a proof-of-concept study, the patient cohort was relatively small, and the patients scanned at 1.5T and 3T had diverse disease etiologies. Therefore, we did not perform statistical comparisons between GB-BOOST at 1.5T and 3T. Although visual comparison based on the preliminary results may indicate that both 2D and 3D images acquired at 1.5T have slightly lower signal-to-noise ratio than those at 3T, more experiments are required to compare the diagnostic performance of GB-BOOST at different field strengths.

Although an isotropic resolution of 1.2 mm was achieved with GB-BOOST, this resolution may be suboptimal for coronary artery imaging, especially for the depiction of small vessels. Improving the resolution would further lengthen the total scan time, making it unfeasible in clinics. Future studies of image acceleration and/or super-resolution with e.g. deep learning-based methods may be helpful to increase spatial resolution and shorten scan time [32], [33].

Lastly, reference 2D breath-held PSIR and 3D CMRA sequences were used as comparison to evaluate the performance of GB-BOOST. Future works may include free-breathing motion-corrected 2D PSIR with signal average as reference, which has better image quality and SNR than the breath-held sequence, particularly for gray-blood LGE generally having a lower SNR than bright-blood LGE. In addition, gold standard references such as invasive coronary angiography and/or histology for CAD and myocardial scar validation may be beneficial in further clinical studies.

## 6. Conclusion

The proposed 3D GB-BOOST sequence can achieve simultaneous whole-heart gray-blood PSIR LGE and CMRA with 1.2 mm isotropic resolution in an efficient scan time of about 10 min. 3D PSIR GB-BOOST LGE achieves accurate scar detection with good contrast between scar, blood, and myocardium, and 3D T2prep GB-BOOST CMRA is comparable with 3D CMRA for the depiction of the coronary arteries. These promising results warrant further studies in a larger patient cohort and comparisons with gold standard references.

## Funding

The authors acknowledge financial support from: (1) BHF programme grant RG/20/1/34802 and King’s BHF Centre for Award Excellence RE/24/130035, (2) Millennium Institute for Intelligent Healthcare Engineering ICN2021_004, (3) Fondecyt 1250261 and Fondecyt 1250252, (4) IMPACT, Center of Interventional Medicine for Precision and Advanced Cellular Therapy FB210024, and (5) the Technical University of Munich—Institute for Advanced Study.

## Author contributions

**Dongyue Si:** Writing – review & editing, Writing – original draft, Methodology, Conceptualization. **Simon J. Littlewood:** Writing – review & editing. **Michael G. Crabb:** Writing – review & editing. **Karl P. Kunze:** Writing – review & editing. **Claudia Prieto:** Writing – review & editing, Supervision, Resources, Project administration, Funding acquisition. **René M. Botnar:** Writing – review & editing, Supervision, Resources, Project administration, Funding acquisition.

## Declaration of competing interests

The authors declare that they have no known competing financial interests or personal relationships that could have appeared to influence the work reported in this paper.
