# Supplementary material for: Comprehensive three-dimensional free-breathing magnetic resonance imaging for simultaneous myocardial viability and coronary artery visualization at 1.5T and 3T
Source: J Cardiovasc Magn Reson. 2025 Dec 12;28(1):102672. doi: 10.1016/j.jocmr.2025.102672 (PMC12811457; doi:10.1016/j.jocmr.2025.102672)
Supplement: Supplementary file 1 — Supplementary material [file mmc1.docx]

**Table S1.** Typical imaging parameters of GB-BOOST at 1.5 T and 3 T.

|  | **1.5 T** | **3 T** |
| --- | --- | --- |
| **FOV (mm^3^)** | 340×340×~100 | |
| **Orientation** | Coronal | |
| **Resolution (mm^3^)** | 1.2 | |
| **Readout** | bSSFP | 2-point Dixon GRE |
| **iNAV** | 14 echos, ramp up | 14 echos, 3° |
| **Acceleration** | 4×VD-CASPR | 5×VD-CASPR |
| **FAs (°)** | 90/90 | 20/10 |
| **TR/TE (ms)** | 3.33/1.46 | 5.23/1.67/3.18 |
| **Bandwidth (Hz/pixel)** | 967 | 827 |
| **T2prep duration (ms)** | 40 | 50 |
| **Lines per readout** | 20~30 | 15~20 |

**
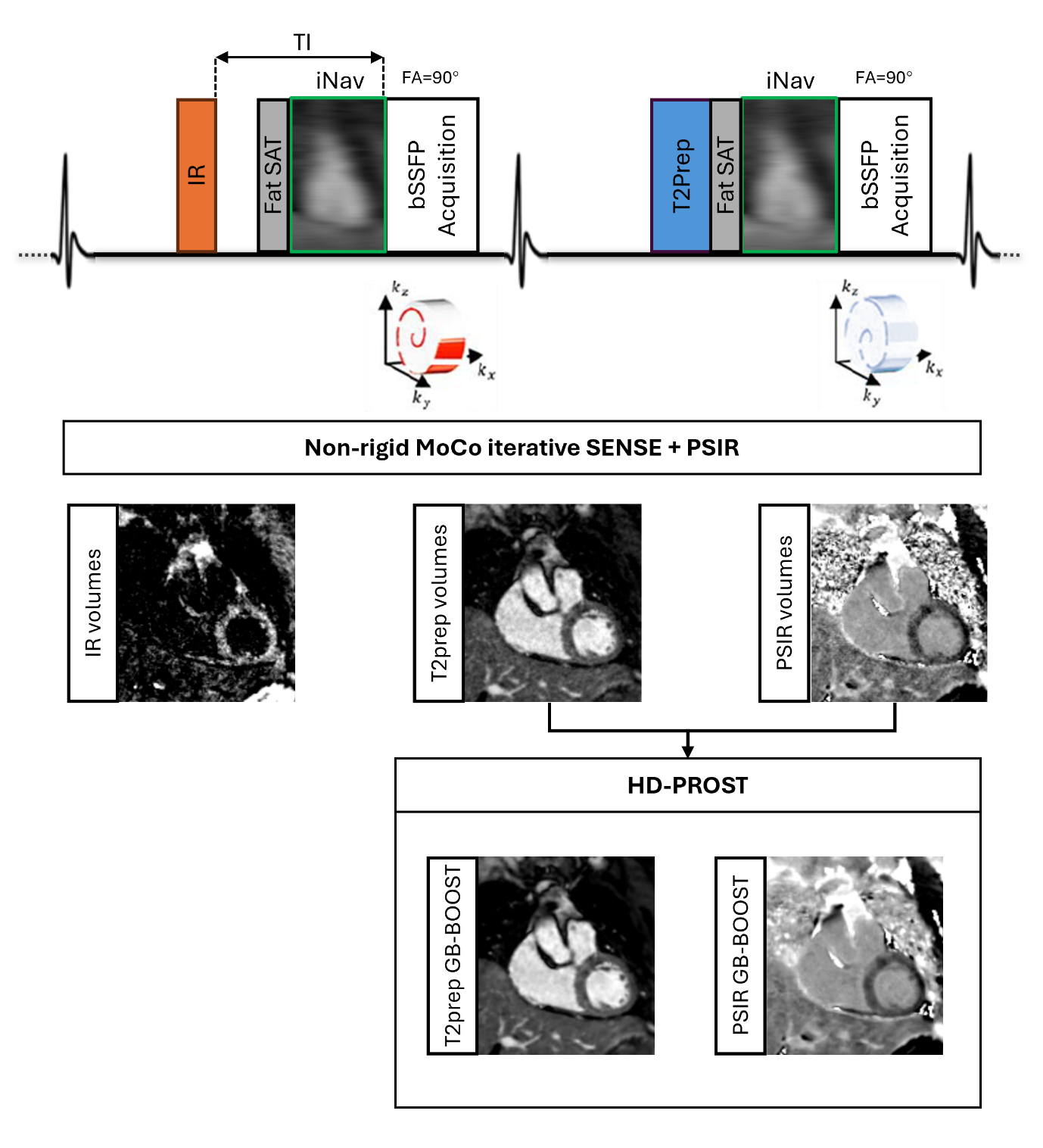
**

**Figure S1.** Sequence and reconstruction framework of the proposed GB-BOOST sequence with balanced steady-state free precession (bSSFP) acquisition. Two electrocardiogram-triggered interleaved 3D volumes are acquired with inversion recovery (IR) and T2 preparation (T2prep) respectively. Inline non-rigid motion corrected (MoCo) iterative SENSE and PSIR reconstruction are performed followed by offline HD-PROST denoising on the T2prep and PSIR volumes to finally generate T2prep GB-BOOST and PSIR GB-BOOST images.

**
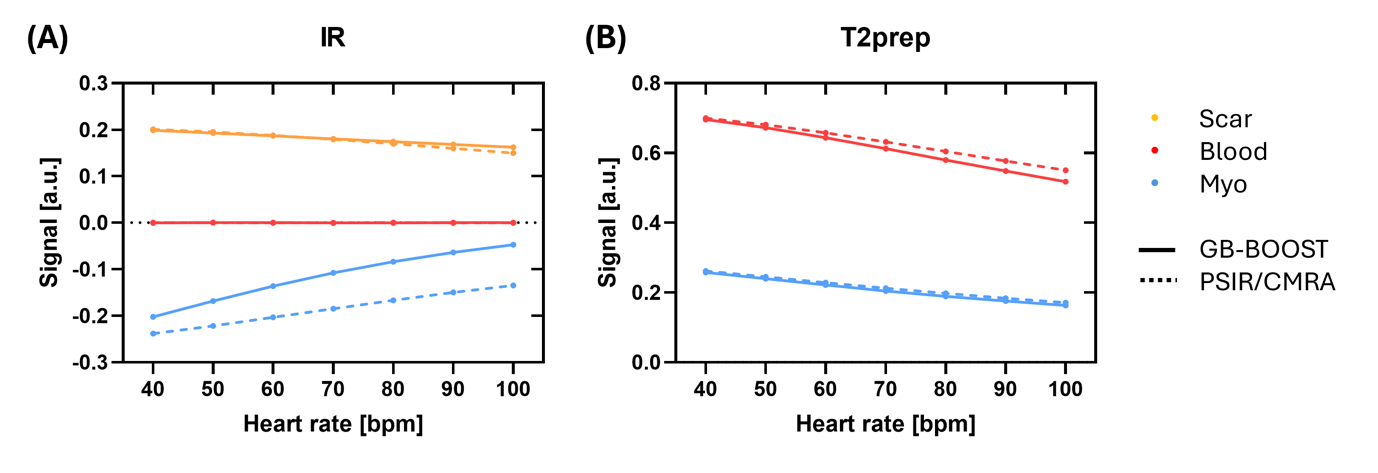
**

**Figure S2.** Simulation results at different heart rate. (**A**) Simulated signal intensity of the IR volume acquired with GB-BOOST and PSIR respectively. (**B**) Simulated signal intensity of the T2prep volume acquired with GB-BOOST and CMRA respectively.

**
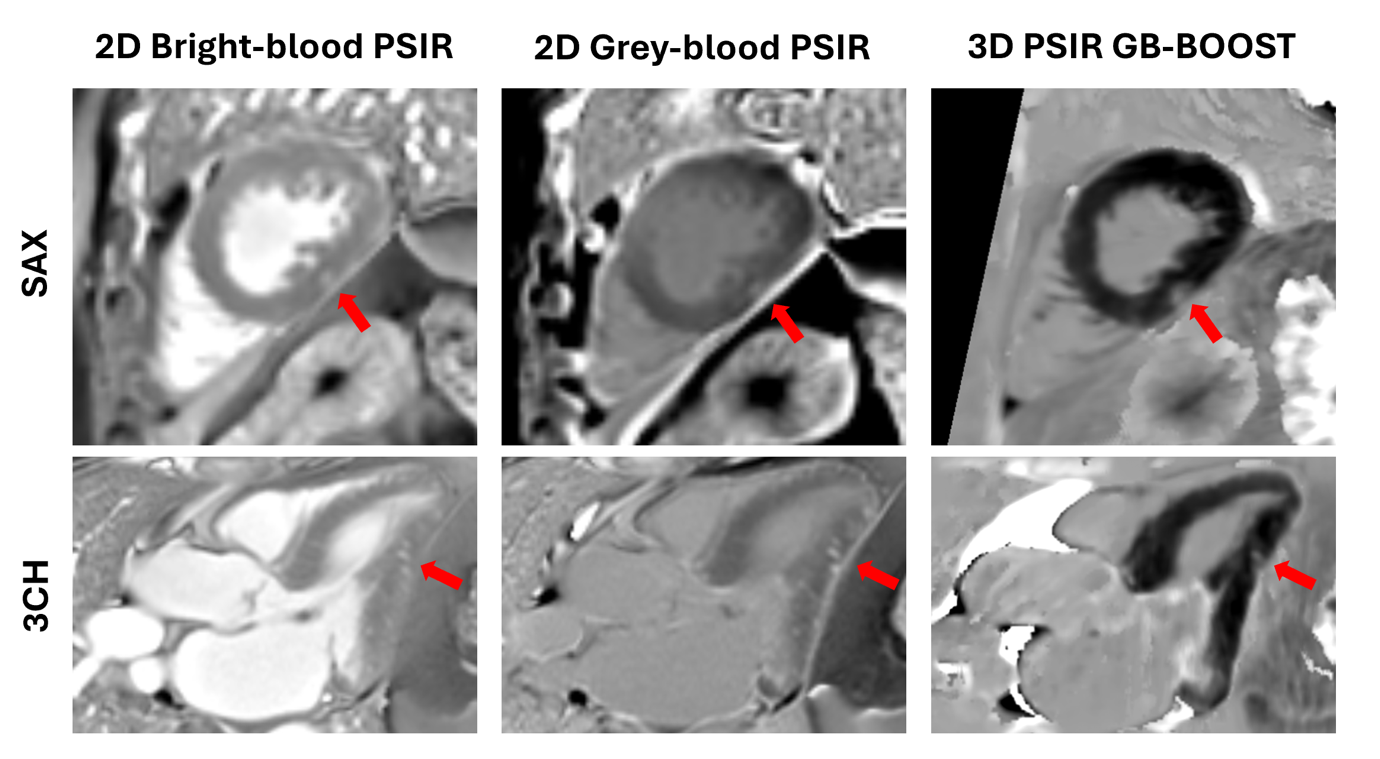
**

**Figure S3.** LGE images of the patient in **Figure 2** acquired with 2D bright-blood PSIR, 2D grey-blood PSIR and 3D PSIR GB-BOOST at 3 T in short-axis (SAX) and 3-chamber (3CH) views. Multiple small scar areas are indicated by red arrows.


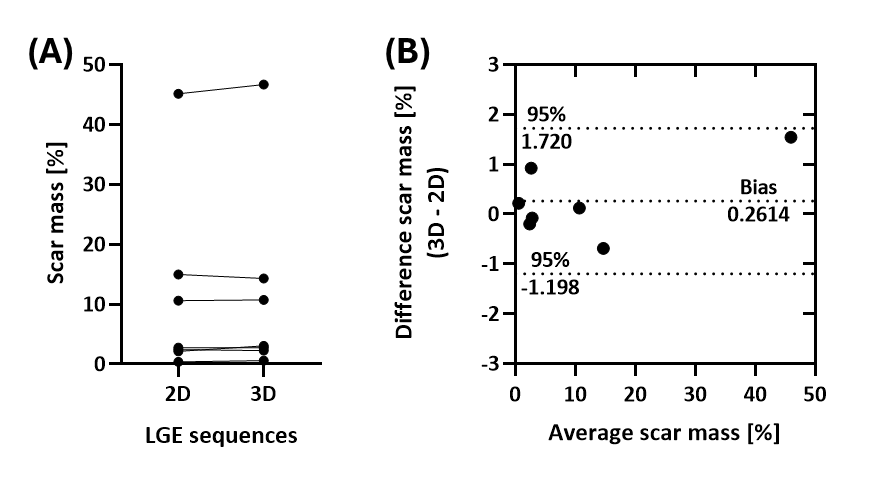


**Figure S4.** Scar mass measured by 2D grey-blood PSIR and 3D PSIR GB-BOOST in the 7 patients with positive LGE findings (**A**) and Bland-Altman analysis of scar mass (**B**).
